# 

**Published:** 1899-02

**Authors:** 


FEBRUARY, 1899.

### THE AMERICAN JOURNAL OF DENTAL SCIENCE.

EDITED BY

F. J. S. GORGAS, M. D., D. D. S.

RICHARD GRADY, M. D., D. D. S.

LIBRARY
SURGEON GENERAL'S OFFICE
FEB. 27 1899

PUBLISHED MONTHLY.

$2.50 PER ANNUM.

Vol. 32. THIRD SERIES No. 10.

BALTIMORE:

SNOWDEN & COWMAN MFG. CO., 9 W. FAYETTE ST.

LONDON:
TRUBNER & CO., 60 PATERNOSTER ROW.

Entered at the Post Office at Baltimore, Md., as Second-class Matter.

PAYABLE IN ADVANCE.

